# Levels of Polycyclic Aromatic Hydrocarbons in the Water and Sediment of Buffalo River Estuary, South Africa and Their Health Risk Assessment

**DOI:** 10.1007/s00244-019-00617-w

**Published:** 2019-03-16

**Authors:** A. O. Adeniji, O. O. Okoh, A. I. Okoh

**Affiliations:** 1 SAMRC Microbial Water Quality Monitoring Centre, University of Fort Hare, Alice, 5700 South Africa; 2 Department of Chemistry, University of Fort Hare, Alice, 5700 South Africa; 3 Applied and Environmental Microbiology Research Group, Department of Biochemistry and Microbiology, University of Fort Hare, Alice, 5700 South Africa

## Abstract

The incidence and spatial distribution of polycyclic aromatic hydrocarbons (PAHs) in the Buffalo River Estuary in the Eastern Cape Province of South Africa were assessed in this study. A total of 60 surface water and 19 sediment samples were collected from 5 sites of the estuary over a period of 6 months (December 2015 to May 2016). Extraction of PAHs from the water and sediment samples was achieved by using liquid–liquid and soxhlet extraction methods respectively, followed by column clean up with silica gel and quantification by gas chromatography–flame ionization detection. Individual PAH levels in the water and sediment samples ranged from not detected (ND) to 24.91 μg/L and ND to 7792 μg/kg, respectively. Total concentrations of the PAHs in the water and sediment samples varied as 14.91–206 μg/L and 1107–22,310 μg/kg in that order. Total levels of the contaminants were above the target values in the two matrices and were higher in summer than autumn. Although the noncarcinogenic risk of PAHs estimated in the water column through dermal absorption was very low compared with the target value, the carcinogenic risk determined was high for both adults and children. Similarly, benzo(a)pyrene and dibenzo(a,h)anthracene were found to be of higher carcinogenic and mutagenic risks in the sediments collected from the study area. Diagnostic ratios suggest that the target hydrocarbons are predominantly from pyrolytic sources. It therefore could be inferred that the water body is conspicuously polluted; hence, efforts should be made to control all the activities contributing to such magnitude of pollution at the sites.

Polycyclic aromatic hydrocarbons (PAHs) are priority organic pollutants, which are ubiquitously found in the atmospheric, aquatic, and terrestrial systems and therefore are closely monitored in the environment (Okoro 2008; Sakuma et al. 2011; Adeniji et al. 2018a). They are multiple ring structures having molecular masses in the range of 128 to 278 Dalton (Kumar et al. 2015). A number of them are genotoxic, mutagenic, carcinogenic, and/or teratogenic in nature with long range of transport and well implicated in endocrine system disruption at levels higher than the maximum allowable limit within a very short time (Wilson et al. 2001; Cai et al. 2009; Brazkova and Krastanov 2013). However, few of the nontoxic PAH congeners are found useful as synergists (ATSDR 1995; Adekunle et al. 2017).

Higher molecular members of this class of pollutants are relatively immobile due to their large molecular volumes and are less volatile, relatively insoluble in water, and more lipophilic than the lower molecular members. They also are known to stay longer in the environment (Wild and Jones 1995; Adeniji et al. 2018a). Their hydrophobicity has generated a lot of concerns amongst the general public because of the accompanying risks to humans, environment, and aquatic organisms. They associate freely with dissolved organic matter in the natural water through several means of binding and adsorption, especially those with high molecular weights (Akkanen et al. 2005; Zheng et al. 2007; Smith et al. 2011; Yu et al. 2011; Lin et al. 2018) and are subsequently deposited in the sediment, thus accumulating to a higher level of toxicity in the aquatic environment (Prabhukumar and Pagilla 2010; Brazkova and Krastanov 2013; Olatunji et al. 2014). The bioavailability of PAHs to the aquatic animals and also their penetration of dietary sources has thus become unavoidable (Sakuma et al. 2011; Adeniji et al. 2018b).

These pollutants are capable of presenting significant health risk to human by oral intake through food, inhalation, and/or even dermal interaction. Exposures through any of the listed routes could bring about health challenges of short- and long-term effects, including some major respiratory and cardiovascular diseases (Perez-Padilla et al. 2010; WHO 2014). Their reactive metabolites, such as epoxides and dihydrodiols, are considered more concerning, given their ability to bind to cellular proteins and DNA and adduct formation (Balbo et al. 2014; Błaszczyk et al. 2017). PAHs are in most cases originated from both natural and anthropogenic origins linked with incomplete combustion of organic materials and release of noncombusted, petrogenic emissions. They enter the environment by way of some natural processes, such as microbial synthesis and volcanic activities, and also through oil exploration and production processes, natural, and intentional burning of biomass and fossil fuels, as well as incomplete combustion of other organic materials including coal (ATSDR 1995; Okoro 2008).

Estuarine and coastal marine environments receive a substantial input of pollutants because of their proximity to land inhabited by humans (Nemcik 2004). Estuaries especially have served as major depositories for the disposal of industrial and domestic effluents, sewage sludge, and dredged material with considerable loads of such contaminants as heavy metals, PAHs, chlorinated hydrocarbons, petroleum hydrocarbons, and radioactive materials coming through pipeline discharges, disposal from vessels, riverine input, vehicular emission, atmospheric deposition, surface runoff, as well as the oil spillage in the aquatic milieus. This makes their presence in the water and sediment matrices a paramount issue with utmost attention for the aquatic environment, fishing, and seafood producing industries (Kennish 1994; Gorleku et al. 2014).

Previous environmental studies at the Buffalo River estuary include assessment of its sediment for heavy metals pollution about two decades ago (RHP 2004) and its water for bacteria load and physicochemical qualities (Chigor et al. 2012, 2013). Those findings revealed gross pollution of both environmental matrices. Recently, fairly high concentrations of total petroleum hydrocarbons, organochlorine pesticides, polychlorinated biphenyls, and some phenolic derivatives also were documented in the zone (Adeniji et al. 2017; Yahaya et al. 2017, 2018, 2019). However, no report is available till date on the status of the polycyclic aromatic hydrocarbons (PAHs) in the area; hence, it became very expedient to investigate, giving its proximity to the river port and some notable industries around. In this paper, the distribution of sixteen (16) priority (PAHs) and their associated health risk in the water and sediment of Buffalo River Estuary, South Africa to humans are reported. Possible sources of the organic contaminants also were predicted using some relevant isomeric ratios.

## Materials and Methods

### Description of the Study Area

**Fig. 1:**
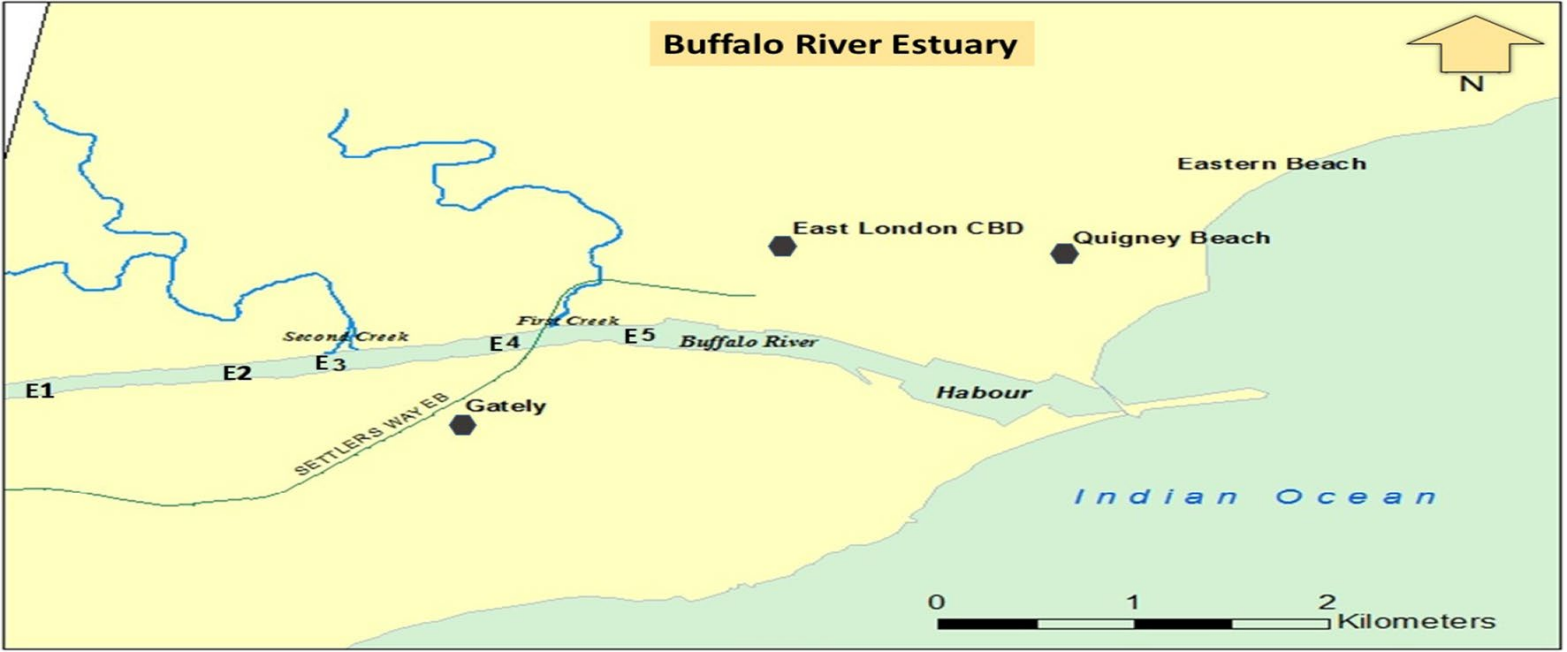
Map of Buffalo River Estuary

Buffalo River Estuary is a large permanently open estuary situated at the East London city in South Africa (DEAT 2010; Whitfield and Baliwe 2013). The 6.5-km-long estuary is found in the downstream of the 126-km-long Buffalo River, which drains into the Indian Ocean (RHP 2004). It is an important estuary among the 139 found in the Eastern Cape Province of South Africa, being the largest River Port in the country, and it plays a major role in the economy of East London (http://soer.deat.gov.za/dm_documents/Theme_Coast_cUAvQ.pdf). It is approximately 2.4–7.4 m deep and covers 98 ha. It is located at the mouth of the long Buffalo River, which flows through some major towns and industrial areas. The Buffalo River carries runoffs, solid wastes, raw sewage, and industrial effluents received from other large rivers into the estuary (RHP 2004). Examples of such influent rivers are the first and second creeks that contribute significantly to the inflow of domestic and industrial wastes into the aquatic environment (EOHCES 2016). Not less than nine wastewater treatment plants are reportedly discharging effluents into the Buffalo River, which greatly impact its pollution level either directly or indirectly (Chigor et al. 2012, 2013). Some direct users around the estuary include East London Port (where shipping activities are carried out daily), BP South Africa, Chevron (Pty) Ltd, Engen, Total South Africa, Vukani Petroleum, Mercedes Benz South Africa, Sea Spirit Fisheries, and many others (Chimuka et al. 2015; EOHCES 2016). A detailed description of the study area presented in Table 1 and Fig. 1 is available in the previous report by Adeniji et al. (2017).

**Table 1:** Description of the Buffalo River Estuary

Study site	Stations	Latitude	Longitude	Depth (m)	Description
Buffalo River Estuary	E1	33.03049°S	27.85821°E	2.40	Receiving point of the Buffalo river influent
	E2	33.02788°S	27.86294°E	3.47	Extension of the Buffalo river influent reception
	E3	33.02612°S	27.88288°E	4.44	Industrial Effluent Discharge Point (Second Creek)
	E4	33.02436°S	27.89072°E	6.16	Under Steve Biko Bridge (First Creek)
	E5	33.02332°S	27.89337°E	7.23	Under Buffalo Bridge

### Reagents, Solvents, and Standards

Dichloromethane, acetone, and hexane (high performance liquid chromatography grade) and n-Pentane (AR grade) used in the processing and analysis of samples were procured from Merck, South Africa. Reagent grade anhydrous sodium sulphate (Merck, South Africa) used as absorbent, silica gel (100–200 mesh) (Radchem Laboratory Supplies, South Africa) for chromatographic column clean-up and concentrated hydrochloric acid were also purchased. *o*-Terphenyl and 2-fluorobiphenyl (used as surrogate standards) and SV Calibration Mix #5 (#31,011) containing 2000 µg/mL each of the 16 priority PAHs also were bought from Restek, USA. Working and surrogate standard solutions were prepared by diluting the stock solutions with HPLC grade n-hexane as required and stored at 4 °C in the dark for approximately 3 months (Dong et al. 2012).

### Sample Collection, Extraction, and Clean-Up

A total of 60 surface water samples were collected early in the morning (between 7 am and 10 am) in duplicates from 5 sampling locations of the Buffalo River Estuary between December 2015 and May 2016 with precleaned glass bottles. The samples in 1-L amber bottles were adjusted to pH < 2 using 6 M of hydrochloric acid, whereas 19 sediment samples (1 kg from each sampling point every month except E5 that was rocky) were collected with Van Veen Grab sampler into wide-mouth bottles. It is worthy of note that water samples were collected all through the 6 months of this research work, but sediment samples were only taken between January and May 2016. Moreover, Site E5 was excluded from sediment sample collection throughout the study period because of its rocky base, whereas E4 was only exempted in May 2016, because no sample was found in spite of several trials. All samples were immediately transported on ice-chest below 4 °C to the laboratory for analysis (Gorleku et al. 2014).

One milliliter of surrogate standard mixture (10 µg/mL) was spiked into each 500-mL aliquot of the water sample and extracted three times with liquid–liquid extraction (LLE) technique using n-hexane as solvent. Extracts were combined, dried over anhydrous sodium sulphate, and concentrated using rotary evaporator to a volume of approximately 2 mL before column clean-up. Sediment samples were air-dried in the dark for approximately 5 days and then were crushed and sieved with 0.5-mm mesh. Approximately 10 g of the sieved sediment sample was mixed with sufficient amount of anhydrous sodium sulphate, spiked with 1 mL of the surrogate standard mixture, and extracted in a Soxhlet extractor with 200 mL of dichloromethane for 24 h. The extract was filtered through anhydrous sodium sulphate into a clean amber glass bottle, concentrated using rotary evaporator and solvent exchanged into n-hexane (WSDE 1997; Kafilzadeh et al. 2011; Adeniji et al. 2018b).

For clean-up, a 10-mm I.D. × 30 cm long chromatographic column was packed with a slurry prepared with 10 g of activated silica gel in dichloromethane. Approximately 2 cm of anhydrous sodium sulphate was added on top of the silica gel to absorb moisture. The column was pre-eluted with 20 mL of n-pentane, and the eluant was discarded. Each sample extract was transferred immediately after pre-elution onto the silica gel column, followed with rinsing of the sample vial and thereafter eluted with 20 mL of n-pentane. This eluant was collected as aliphatic fraction, and the aromatic fraction was then eluted with 40 mL of dichloromethane/pentane (40:60) (v/v), concentrated, and solvent exchanged into dichloromethane (WSDE 1997; Kafilzadeh et al. 2011; Kumar et al. 2014a). The moisture, organic carbon, and organic matter contents of the sediment samples were gravimetrically determined in accordance with the methods of Motsara and Roy (2008) and Olutona et al. (2016).

### Analysis of Polycyclic Aromatic Hydrocarbons

Agilent 7820A gas chromatograph with HP-5 fused silica capillary column (30 m × 0.320 mm i.d. × 0.250-μm film thickness) and flame ionisation detector was used for the quantification of the analytes of interest. Sample of 1-μL volume was injected in splitless mode at a temperature of 280 °C. The oven temperature was programmed to start from 70 °C (3 min) to 325 °C at 12 °C/min (6 min). The carrier gas was helium (99.999%) at average velocity and flow rate of 29.449 cm/sec and 1.6255 mL/min, respectively. FID temperature was 300 °C, and other detector parameters used are summarised as follows: H_2_ flow rate: 45.0 mL/min; air flow rate: 450 mL/min and N_2_ flow rate: 20 mL/min. Calibration standards were prepared in the working range of 0.05–20 μg/mL by serial dilution with n-hexane (Olatunji et al. 2014). The peak areas calculated using the baseline–baseline mode with Agilent Chemstation software were used to plot calibration curves for all the 16 PAH congeners (Ma et al. 2010; Nekhavhambe et al. 2014). The curves were all linear with correlation coefficients falling within the acceptable range of r^2^ ≥ 0.990 (Adeniji et al. 2017). Samples were thereafter identified by their retention times, whereas quantification was done by the instrument on the basis of the response factor generated for each congener from the linear curve plotted (Ma et al. 2010; Nekhavhambe et al. 2014).

### Probable Carcinogenic and Mutagenic Toxicities of PAHs in the Estuary Sediment

Potential carcinogenic and mutagenic toxicities of the high molecular weight PAHs detected in the sediment samples collected from the study locations were assessed relative to benzo[a]pyrene (with sufficient toxicological information) using toxic equivalent quotient (TEQ) and mutagenic equivalent quotient (MEQ) as shown in Eqs. (1) and (2), respectively.

1 $${\text{TEQ}} = \varSigma C_{n} \cdot {\text{ TEF}}_{n}$$

2 $${\text{MEQ}} = \varSigma C_{n} \cdot {\text{MEF}}_{n}$$

where *C*_*n*_ = concentration of each PAH congener (*n*) in the mixture. TEF_*n*_ = toxic equivalence factor (TEF) for each PAH congener (*n*). MEF_*n*_ = mutagenic equivalent factor (MEF) for each PAH congener (*n*). The TEF values used for BaA, Chry, BbF, BkF, BaP, DiahA, Inpy, and BghiP in these calculations were 0.1, 0.01, 0.1, 0.1, 1, 1, 0.1, and 0.01, respectively, and their corresponding MEF values were 0.082, 0.017, 0.25, 0.11, 1.0, 0.29, 0.31, and 0.19 (Van den Berg et al. 2006; CCME 2010; Lerda 2011; Benson et al. 2017; Zhao et al. 2017).

### Health Risk Assessment to Humans in the Estuary Water

In this study, health risk to humans was assessed by calculating the potential carcinogenic and noncarcinogenic effects of exposure of a person to the PAHs over a certain period of time (Gerba 2006; US EPA 2001, 2009; Titilawo et al. 2018). The evaluation was done on the basis of exposure by dermal contact only since the waterbody is a recreational environment (EOHCES 2016).

Average daily dosage by dermal contact (ADD_derm_) expressed in mg/kg/day was calculated for noncarcinogenic risks connected with PAHs in the water column of the study area as shown in Eq. (3).

3 $${\text{ADD}}_{\text{derm}} = \frac{{C \times {\text{SA}} \times {\text{Kp}} \times {\text{ET}} \times {\text{EF}} \times {\text{ED}} \times {\text{CF}}}}{{{\text{BW}} \times {\text{AT}}}}$$

where ADD_derm_ represents the average daily doses by dermal contact (mg/kg/day); *C* stands for the concentration of PAHs in the water sample (mg/L); EF is the exposure frequency (350 days/year for both ingestion and dermal absorption); ED is the exposure duration (adult: 30 years; child: 6 years); BW represents the average body weight (adult: 70 kg; child: 15 kg); AT means the average time, i.e., ED × 365 days (adult: 10,950 days; child: 2190 days); SA stands for the exposed skin area (adult: 18,000 cm^2^; child: 6600 cm^2^); Kp (cm/h) is the dermal permeability coefficient (corresponding values are shown in Tables 4 and 5); ET is the exposure time of shower and bathing (adult: 0.58 h/day; child: 1 h/day); and CF represents the unit conversion factor (*L*/1000 cm). Guidelines values provided by Department of Environmental Affairs, South Africa, and US EPA were used for the estimations (US EPA 1989, 1992; Gerba 2006; DEA 2010; DTSC 2014; Feng et al. 2016a; Wang et al. 2018).

For noncarcinogenic PAHs, hazard quotient (HQ) was calculated by multiplying the ADD with reference dose (RfD) for individual contaminant as presented in Eq. (4). Hazard index (HI) and the sum of HQs also was estimated for all PAH congeners in the samples using Eq. (5) (US EPA 1989; Wei et al. 2015).

4 $${\text{HQ}} = \frac{{\text{ADD}}}{{\text{RfD}}}$$

5 $${\text{HI}} = \sum {\text{HQs}}$$

RfD, which represents dermal reference dose for each PAH congener, was not available for other priority compounds except for only six: i.e., naphthalene, fluorene, anthracene, phenanthrene, fluoranthene, and benzo(g,h,i)perylene (Table 4).

Moreover, incremental lifetime cancer risk (ILCR) and risk index (RI) were determined for cPAHs found in the water samples using LADD (mg/kg/day), in which Eq. (1) was used to calculate the LADD (by dermal contact) the same way as for ADD, only that AT = 25,550 was used for the two age categories instead of the previous value. No Kp value was available for BkF; hence, LADD_derm_ and ILCR_derm_ were not computed for it.

For the calculation of ILCR and RI in the water samples, Eqs. (6) and (7) were used according to US EPA guidelines (USEPA 1989, 2015; Jamhari et al. 2014).

6 $${\text{ILCR}} = {\text{LADD}} \times {\text{CSF}}$$

7 $${\text{RI}} = \sum {\text{ ILCR}}$$

where CSF represents the cancer slope factor for each PAH congener. CSF for BaP is 7.3 mg/kg/day (US EPA 2015). Factors for other PAHs were afterward calculated from the values for BaP by taking a multiple of TEF for each compound and its respective CSF as presented in Table 5 (IARC 2006; Kumar et al. 2015; Wei et al. 2015).

### Quality Control and Quality and Assurance

All of the glass apparatus used were washed and rinsed with tap and distilled water successively. They were subsequently dried in the air circulated oven, cooled, and rinsed with acetone (Dong et al. 2012). Methods blanks were determined alongside the samples in duplicates, and their values were generally below the detection limit (Gorleku et al. 2014; Kumar et al. 2014a). Limits of detection and quantification for all the selected contaminants ranged from 0.01 to 0.03 μg/L and 0.04 to 0.15 μg/L, respectively, whereas their relative standard deviations varied between 0.59% and 2.51%. Concentrations below detection limits were recorded as zero in calculation but reported as not detected (Jiao et al. 2012; Gorleku et al. 2014; Olatunji et al. 2014; Kumar et al. 2015). The overall mean recovery of PAHs in both water and sediment samples were found to be 79.53% and 72.20%, correspondingly in agreement with literatures (US EPA 2003; Kelly et al. 2000; Gorleku et al. 2014). Likewise, the recoveries of surrogate standards were within the acceptable range and so were used to correct the concentrations of the analytes of interest in the samples (Mirza et al. 2012; ESS Laboratory 2008; KDHE 2015).

### Statistical Analyses

Descriptive statistics and analysis of variance were carried out using stataIC 12 (64-bit) to assess means and standard deviations of data, as well as the disparity between and within them in groups. The *p* value < 0.05 was considered significant cutoff.

## Results and Discussion

### Levels of PAHs in the Water Samples from Buffalo River Estuary

The levels of PAHs in the water column of Buffalo River Estuary are given in Table 2. All 16 priority PAHs of the United States Environmental Protection Agency (US EPA) were detected in the water samples collected from the estuary at varied concentrations, confirming the ubiquitous nature of the pollutants (Jiao et al. 2012; Wang et al. 2018). Individual levels of PAHs in the water phase ranged from nondetected (ND) to 24.91 μg/L (indeno(1,2,3-cd)pyrene). The most and least frequently detected of the pollutants were benzo(a)anthracene (90%) and acenaphthylene (7%), respectively. As shown in Table 2, naphthalene, chrysene, and benzo(a)pyrene (a group 1 carcinogen) exceeded their maximum allowable concentrations (MAC) in fresh and marine waters (British Columbia 1993). Permissible limit of 0.2 μg/L set by Agency for Toxic Substances and Disease Registry (ATSDR 2009) for benzo(a)pyrene also was exceeded. The total concentrations of the 16 PAHs (∑PAHs) varied in water from 14.91 to 206 μg/L with a mean value of 76.06 ± 11.01 μg/L (Table 2). Guidelines from other countries were used in this study, because no limit is at the moment set in South Africa for PAHs in different compartments of environment (Chimuka et al. 2015).

**Table 2:** Concentrations of the 16 priority PAHs in the water and sediment samples from Buffalo River Estuary

PAHs	Surface water				Sediment				
	Range (µg/L)	Mean (µg/L)	MAC (µg/L)	FD (%)	Range (µg/kg)	Mean (µg/kg)	ERL (µg/kg)	ERM (µg/kg)	FD (%)
Naphthalene	ND—12.69	6.94 ± 0.6	1	17	ND—652	361 ± 40.2	160	2100	42
Acenaphthylene	ND—14.29	7.39 ± 1.78	NR	7	ND—816	119 ± 43.28	44	640	84
Acenaphthene	ND—19.03	3.05 ± 1.29	6	23	ND—731	177 ± 61.11	16	500	74
Fluorene	ND—12.11	3.59 ± 0.50	12	63	ND—1380	435 ± 96.4	19	540	95
Anthracene	ND—7.81	1.97 ± 0.32	NR	47	ND—749	230 ± 59.41	853	1100	95
Phenanthrene	ND—14.22	3.59 ± 0.76	NR	47	ND—476	173 ± 33.24	240	1500	95
Fluoranthene	ND—0.54	0.24 ± 0.04	NR	13	ND—456	109 ± 28.66	600	5100	74
Pyrene	ND—8.72	1.32 ± 0.35	NR	60	ND—923	125 ± 50.24	665	2600	95
Benzo(a)anthracene	ND—10.34	3.24 ± 0.45	NR	90	56.94—1490	223 ± 73.36	261	1600	100
Chrysene	ND—19.01	4.39 ± 0.82	0.1	87	ND—1015	398 ± 67.47	384	2800	95
Benzo(b)fluoranthene	ND—7.41	3.52 ± 0.37	NR	70	33.49—1107	325 ± 81.7	NA	NA	100
Benzo(k)fluoranthene	ND—9.26	3.12 ± 0.41	NR	60	ND—772	205 ± 40.65	NA	NA	79
Benzo(a)pyrene	ND—6.25	3.29 ± 0.31	0.01	73	ND—351	185 ± 21.48	430	1600	90
Dibenzo(a,h)anthracene	ND—19.34	11.41 ± 0.95	NR	43	ND—2799	827 ± 167	63.4	260	79
Indeno(1,2,3-cd)pyrene	ND—24.91	7.2 ± 1.03	NR	67	ND—7792	712 ± 420	NA	NA	79
Benzo(g,h,i)perylene	ND—20.26	11.79 ± 1.03	NR	47	ND—801	456 ± 33.76	NA	NA	79
∑PAHs	14.91–206	76.06 ± 11.01		–	1107–22,310	5060 ± 1319	4000	44,792	–
∑LMW	ND—53.24	26.53 ± 5.25		–	37.45—2538	1496 ± 334	–	–	–
∑HMW	ND—90.56	49.53 ± 5.76		–	383—9747	3564 ± 985	–	–	–
∑cPAHs	6.66—96.53	36.17 ± 4.34		–	674—15,326	2875 ± 872	–	–	–

*∑PAHs* sum of polycyclic aromatic hydrocarbons, *∑cPAHs* sum of carcinogenic polycyclic aromatic hydrocarbons, *∑LMW* sum of low molecular weight PAHs, *∑HMW* sum of high molecular weight PAHs, *FD* frequency of detection, *MAC* maximum allowable concentrations, *ERL* effects range low, *ERM* effects range median, *NR* not recommended (British Columbia 1993; Jiao et al. 2012)

**Fig. 2:**
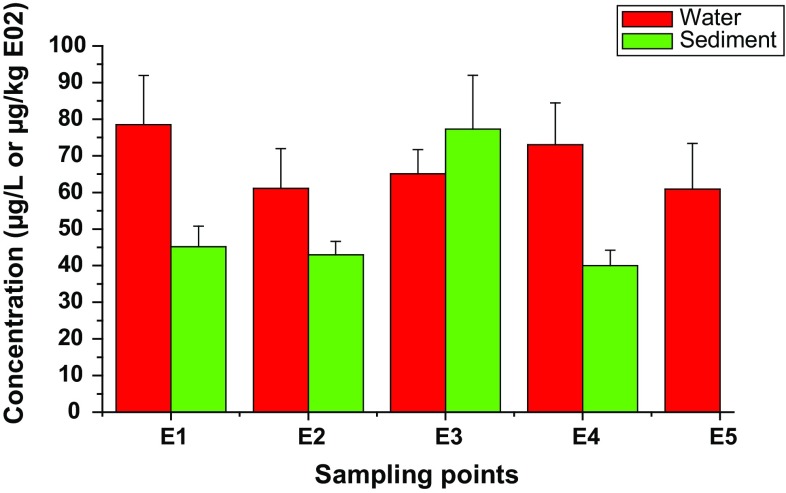
Spatial variability of PAHs in the Buffalo River Estuary

Highest total concentration of PAHs (78.55 μg/L) in the estuary waters was observed at E1 (the shallow entry point of the Buffalo River water into the estuary), followed by E4 (73.03 μg/L) and E3, second creek (65.06 μg/L). The levels at E1 may be attributed to pollution load along the course of the river from major towns, such as Zwelisha, King Williams Town, and Mdantsane, whereas the pollution at E4 may be related to probable leakage of petroleum products from two-stroke engines of the fishing boats, which are usually parked at the Fish Market in the area, stormwater from East London harbour, vehicular emissions from Steve Biko Bridge, and nonpoint source pollution that enters the waterbody as runoff and sewerage through the first creek (RHP 2004; EOHCES 2016). Similarly, PAHs’ contamination of water at E3 cannot be unconnected with the discharge of industrial wastewater and accumulation of leachates at the creek possibly from an old solid waste landfill site, wastewater treatment facilities, and Wilsonia industrial and residential areas (Adeniji et al. 2017). Concentrations of the organic contaminants in all the sampling points were higher than the target value of 30 μg/L for PAHs in the marine waters (Fig. 2) (DoE 2003).

**Fig. 3:**
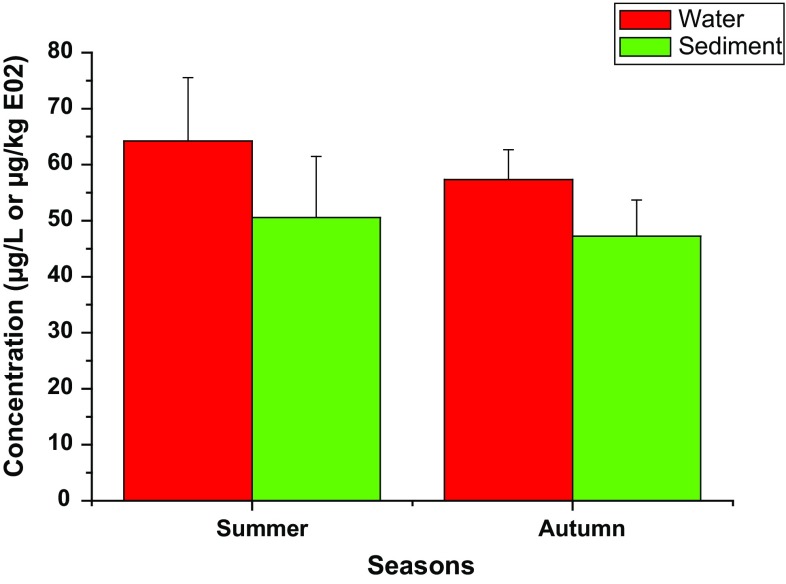
Seasonal concentrations of PAHs in Buffalo River Estuary

The amount of the congeners recorded in summer (64.21 μg/L) was higher than in autumn (57.33 μg/L) as shown in Fig. 3. This may be linked with increasing runoff in the season through which contaminants were possibly swept into the aquatic environment (Kumar et al. 2014a; Adeniji et al. 2017). The total mean concentrations of PAHs in the water compartment of Buffalo River Estuary were above the levels found in the middle and lower reaches of the Yellow River, China (Li et al. 2006), comparable to those reported for Ekpan Creek, Warri, Nigeria (Okoro 2008), and Tema Harbour, Ghana (Gorleku et al. 2014) and were below those found in some major rivers in Limpopo Province, South Africa (Nekhavhambe et al. 2014; Edokpayi et al. 2016).

### Levels of PAHs in the Sediment Samples from Buffalo River Estuary

The concentrations of the 16 PAHs of concern in the surface sediment of Buffalo River Estuary, expressed on dry weight (dw) basis are given in Table 2. Benzo(a)anthracene (100%) and benzo(b)fluoranthene (100%) were the most detected of all, whereas naphthalene (42%) was the least. Individual PAHs in the sediment samples were found with concentrations in the range of ND to 7792 μg/kg (indeno(1,2,3-cd)pyrene). Total concentrations, calculated as sum of the 16 PAHs in the estuary sediment varied from 1107 μg/kg to 22,310 μg/kg, having an average of 5060 ± 1319 μg/kg which was much higher than the effect range low (ERL) of 4000 μg/kg. Naphthalene, acenaphthylene, acenaphthene, fluorene, chrysene, and dibenzo(a,h)anthracene also were determined at levels higher than their respective ERLs as shown in Table 2, which suggest that the aquatic organisms, especially the benthic invertebrates in the water milieu might suffer possible adverse biological effects (MacDonald et al. 2000). It worthy to note that the level of benzo(a)pyrene in the estuary sediment was below its ERL value of 430 µg/kg (Dong et al. 2012; Jiao et al. 2012).

Sediment samples were collected only in the first four sampling points of the study site, because the last point under Buffalo Bridge (E5) was rocky; hence, no sample was collected from there throughout the lifetime of this work. The distribution of PAHs in the estuarine sediment revealed that second creek (E3) was the most polluted (7728 µg/kg dw), followed by E1, the shallow entry point of Buffalo River (4515 µg/kg dw) as shown in Fig. 2. The fairly low level of ∑PAH recorded at E4 may be ascribed to the possible tidal flushing of contaminants adsorbed on sediment particle close to the mouth of the estuary where it discharges into the Indian Ocean (Zhao et al. 2017). Average levels at the four sampling points were all above the ERL and below effect range median (ERM; 44,792 µg/kg dw). They were found within the intermediate range of ERL–ERM, suggesting occasional adverse biological effects to the aquatic animals. Concentrations above ERM value is an indication of adverse effects on the ecosystem and likely danger to human health as well (Jiao et al. 2012; Tornero and d’Alcalà 2014; Adeniji et al. 2018a, b). The concentrations recorded in this study therefore imply that the estuary is fairly polluted with PAHs. Possible sources of PAHs in the estuary, especially at first and second creeks, could be related to effluent from automobile and petrochemical industries contaminates in the area, leachate from the nearby landfill site, West Bank Hood Point domestic and industrial wastewater channels, vehicle emissions on the highways, and urbanised subcatchments’ drains from the East London City Centre (RHP 2004; Okoro 2008; Kafilzadeh et al. 2011; Sule et al. 2011; EOHCES 2016).

The percentage organic carbon (OC) in the sediment samples ranged from 5.17% (E4, first creek) to 6.78% (E2, Buffalo River influent) with a mean value of 6.14%. The relationship between the  %OC and total concentration of PAHs in the sediment samples was assessed and the Pearson correlation obtained was though positive but weak (r = 0.469, *p* < 0.1), implying that organic carbon content has little contribution to the sorption of PAHs to the estuary sediment (Jiao et al. 2012). Therefore, OC and PAHs found in the sediments might be from different origins and might as well be affected by dissimilar biogeochemical processes (Kang et al. 2009). Assimilation of organic and inorganic wastes in the sediment possibly from occasional leakage of oil from the harbour and other port activities in the estuarine zone, as well as continuous discharge of industrial effluents into the waterbody could to an extent be contributory the level of organic carbon recorded (EOHCES 2016). High levels of OC in the estuary can greatly influence the sorption, desorption, and/or biodegradation of PAHs, and as well determine their level of accumulation in the sediment subsequently, causing great harm to the aquatic organisms in the ecosystem (Prabhukumar and Pagilla 2010; Jiao et al. 2012; Brazkova and Krastanov 2013). Concentrations of PAHs determined in summer (5056 µg/kg) were higher than in autumn (4724 µg/kg), which also was the case with the water samples (Fig. 3). This might result from strong rainfall in the season, possibly carrying large amount of the contaminants into the waterbody in the form of runoff from the highways and drainages of the urban city of East London and industrial areas in the neighbourhood (Li et al. 2017). Comparatively, the levels of PAHs obtained in the Buffalo River Estuary sediments were similar to those from Casco Bay, Maine, Texas (Kennicutt II et al. 1994), Sydney Harbour (Montoya 2015), Delhi, India (Kumar et al. 2014a), Xinxiang, China (Feng et al. 2016b), major rivers in Limpopo Province, South Africa (Edokpayi et al. 2016), and marine environment, Korea (Yim et al. 2005) but were higher than the concentrations reported for middle and lower reaches of the Yellow River, China (Li et al. 2006).

### Ring sizes, Diagnostic Ratios, and Possible Sources of PAHs in the Estuary

**Fig. 4:**
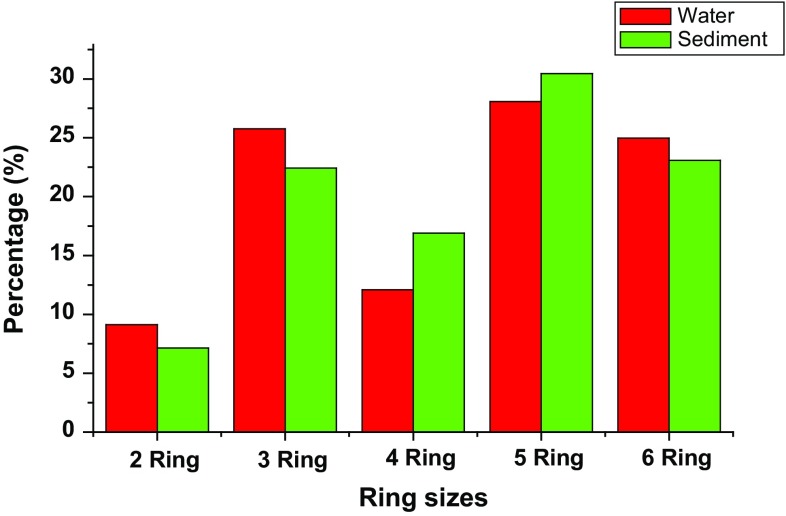
Relative abundance of PAHs in the Buffalo River Estuary

Ring size distribution followed a decreasing order 5 > 3 > 6 > 4 > 2 in water samples and 5 > 6 > 3 > 4 > 2 in the sediment samples (Fig. 4). This shows that 5 rings PAHs were the most abundant in both environmental matrices, although the water samples were distinguished with higher percentage of 3 rings compounds, whereas 6 rings PAHs were more abundant in the sediment samples (Nekhavhambe et al. 2014). Distribution of high molecular weight PAHs (HPAHs) and low molecular weight PAHs (LPAHs) were calculated to identify the possible sources of PAHs pollution in the estuary. The ratio ΣLMW/ΣHMW was lower than 1 in all the sampling points (Table 3), meaning that the concentration of the HPAHs (which are more toxic) was generally higher in the environment than the LPAHs, suggesting a dominant pyrolytic input rather the petrogenic sources in both water and sediment matrices (Hasanati et al. 2011; Kafilzadeh et al. 2011; Onojake et al. 2014). It is understandable because the LPAHs are characteristically volatile and more biodegradable, unlike the HPAHs which are more persistent in the environment (Doan 2005).

**Table 3:** Molecular diagnostic ratios and possible sources of PAHs in the water and sediment samples from Buffalo River Estuary

PAHs	Petrogenic	Pyrolytic	Surface water	Sediment
Anth/178	< 0.1	≥ 0.1	0.01 ± 0.001	1.22 ± 0.33
Anth/Anth + Phen	< 0.1	> 0.1	0.5 ± 0.07	0.44 ± 0.06
BaA/228	< 0.2	0.2–0.35	0.01 ± 0.002	0.98 ± 0.32
BaA/BaA + Chry	< 0.2	0.2–0.35	0.52 ± 0.05	0.41 ± 0.06
Chry/BaA	< 0.4	> 0.9	2.19 ± 0.62	3.09 ± 0.69
Flt/Pyr	< 1.0	> 1.0	0.05 ± 0.03	1.67 ± 0.56
Flt/Flt + Pyr	< 0.4	> 0.4	0.04 ± 0.02	0.4 ± 0.09
InPy/InPy + BghiP	< 0.2	> 0.2	–	0.47 ± 0.06
LMW/HMW	> 1.0	< 1.0	0.54 ± 0.91	0.42 ± 0.34
Phen/Anth	> 15	< 10	0.61 ± 0.12	2.38 ± 0.91

*Phen* phenanthrene, *Anth* anthracene, *Chry* chrysene, *BaA* benzo[a]anthracene, *Flt* fluoranthene, *Pyr* pyrene, *InPy* indeno(123,cd)pyrene, *BghiP* benzo[g,h,i]perylene, *LMW* low molecular weight, *HMW* high molecular weight (Moyo et al. 2013; Adeniji et al. 2018a, b)

Other diagnostic ratios of specific PAH congeners and their isomers have been used as geochemical tracers and also to distinguish natural and anthropogenic sources of PAHs in urban and rural areas (Mirza et al. 2012). Ten of such ratios were in all selected for use in this study (Table 3). Approximately eight of them pointed to pyrolytic origins in the sediment, whereas only four agreed with that prediction in the water phase. Another four diagnostic ratios (Anth/178, BaA/228, Flt/Pyr, and Flt/Flt + Pyr) in the water column, however, suggested predominant petrogenic sources of PAHs in the area (Douglas et al. 2007; Hasanati et al. 2011; Kafilzadeh et al. 2011; Sule et al. 2011). The inconsistence of these ratios was not unexpected because of their possible instability, which has been reported previously (Sany et al. 2014; Saha et al. 2009; Jiang et al. 2009; Adeniji et al. 2018a, b). Notwithstanding, petrogenic diagnosis in the estuary water may be attributed to possible leakage of fuel from two-stroke engines of the fishing boats and yacht in the area (Nukpezah 2010; Gorleku et al. 2014). BaA/BaA + Chry is more peculiar among the ratios used. Values < 0.2 usually indicate petrogenic origin, between 0.2 and 0.35 points to combustion of petroleum products, whereas combustion of wood, grass, and/or coal will be suspected if higher than 0.35. Table 3 shows values > 0.35 for both water and sediment, implying contributions from the burning of nonpetroleum materials, such as refuse or biomass as another major pyrolytic input. This assertion was confirmed in the sediment samples with the use of BaA/228 (Jiao et al. 2012; Zhao et al. 2017).

The overall assessment of the result points to a predominant pyrogenic contamination of the two matrices in the aquatic environment (Menzie et al. 2002; Okoro 2008), largely due to influx of runoff from the highways, discharge of treated or untreated effluents, air deposition or incomplete combustion of biomass (wood, plants, domestic wastes) deposited on a dumpsite in the surroundings, and/or stormwater from the nearby port activities (Doan 2005; Kafilzadeh et al. 2011; Sule et al. 2011; Nekhavhambe et al. 2014; Błaszczyk et al. 2017).

### Probable Carcinogenic and Mutagenic Toxicities of PAHs in the Estuary Sediment

Concentrations of the 7 PAHs (BaA, Chry, BbF, BkF, BaP, DiahA, and InPy) with probable carcinogenic potentials (Lerda 2011) ranged from 674 to 15,326 µg/kg dw in sediment samples (mean concentration of 2875 ± 872 µg/kg dw). The carcinogenic PAHs (cPAHs) accounted for 56.82% of the total concentrations of the 16 priority PAHs in the sediment samples (Table 2). The highest concentration of cPAHs in the sediment was recorded from second creek, E3 (4073 µg/kg), suggesting that industrial effluent discharges and leachate from landfill site contribute hugely to the pollution of the estuary. Moreover, indeno(1,2,3-c,d)pyrene, a reliable indicator of incomplete combustion of PAHs was the highest cPAH detected in the sediment samples, indicating that emission from vehicle exhausts is another major pollution source in the study area (Stogiannidis and Laane 2015).

**Fig. 5:**
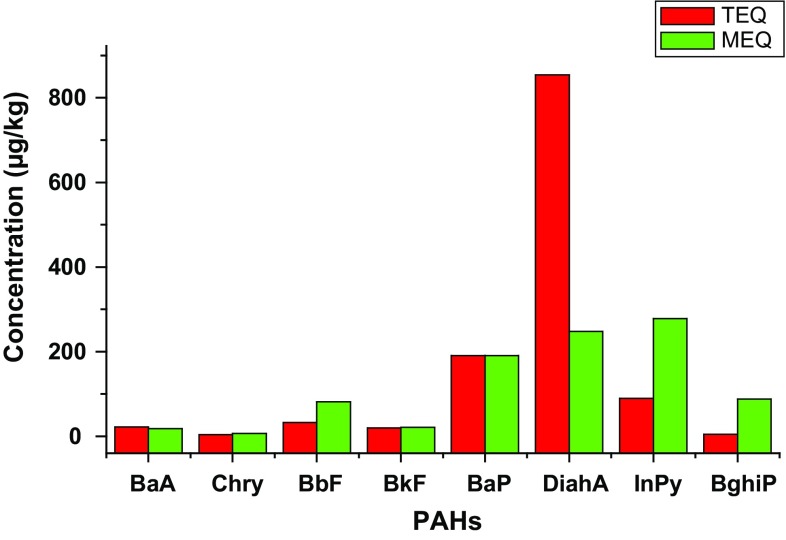
Ecological risk assessment of PAHs in Buffalo River Estuary

The evaluated potential carcinogenic (TEQ) and mutagenic (MEQ) toxicities of PAHs in the estuary sediment are shown in Fig. 5. The total TEQ obtained was 1213 µg/kg, of which BaP (191 µg/kg) and DiahA (854 µg/kg) made significant contributions of 11% and 70%, respectively. This was very consistent with the account of Kumar et al. (2014b). Likewise, the overall MEQ recorded in this study was 932 µg/kg, predominated with BaP (191 µg/kg), DiahA (248 µg/kg), and InPy (278 µg/kg), in a similar manner as reported by Benson et al. (2017). The large influence of BaP and DiahA in both TEQ and MEQ revealed that exposure to the estuary sediment can possibly generate cancer risk and some other noncancer-based health issues that can negatively impact upon the DNA (deoxyribonucleic acid) in humans (Salem et al. 2014; Hussein et al. 2016; Benson et al. 2017; Błaszczyk et al. 2017).

### Assessment of Health Risk to Humans in the Estuary Water

PAHs bioconcentrate and bio-accumulate in organisms through inhalation, ingestion, and/or dermal contact route(s) (Kumar et al. 2014b; Adeniji et al. 2018a, b). The health risk in the Buffalo estuary water was assessed using dermal absorption pathway, being a recreational environment used for several activities, including yacht and rowing clubs. It also is believed to serve as nursery ground for marine invertebrates and fish, although yet to be proven (EOHCES 2016).

The estimated hazard quotients of PAHs in the estuary water by dermal absorption ranged from 9.11 × 10^−4^ to 1.02 × 10^−2^ and from 3.09 × 10^−4^ to 3.47 × 10^−3^ in children and adults, respectively (Table 4). The results indicate that HQs for both age categories in the water column were below the US EPA maximum limit of 1. Hence, the chance of humans contracting any noncarcinogenic disease or other health-related issues by using the water for recreational purposes is very unlikely (ATSDR 2013; Wei et al. 2015; Benson et al. 2017). However, the dermal incremental lifetime cancer risk values calculated were higher. ILCRs varied between 6.94 × 10^−6^ and 9.87 × 10^−4^ for children and from 1.18 × 10^−5^ to 1.67 × 10^−3^ for adults, respectively, with chrysene and benzo(a)pyrene recording the lowest and highest values in that order (Table 5). The risk indices for the two groups were 1.97 × 10^−3^ and 3.34 × 10^−3^, correspondingly. In particular, 5 of the 7 cPAHs, which are benzo(b)fluoranthene, benzo(k)fluoranthene, benzo(a)pyrene, dibenzo(a,h)anthracene, and indeno(1,2,3-cd)pyrene exceeded the threshold value of 1 × 10^−4^ (Wei et al. 2015; Wang et al. 2018).

**Table 4:** HQs of PAHs in the surface water samples from Buffalo River Estuary by dermal contact

PAHs	Kp (cm/h)	RfD	Children	Adults
Naphthalene	6.90 × 10^−2^	0.02	1.01 × 10^−2^	3.43 × 10^−3^
Fluorene	–	0.04	–	–
Anthracene	–	0.04	–	–
Phenanthrene	2.70 × 10^−1^	0.04	1.02 × 10^−2^	3.47 × 10^−3^
Fluoranthene	3.60 × 10^−1^	0.04	9.11 × 10^−4^	3.09 × 10^−4^
Benzo(g,h,i)perylene	–	0.04	–	–
HI			2.13 × 10^−2^	7.2 × 10^−3^

*PAHs* polycyclic aromatic hydrocarbons, *RfD* reference dose, *HI* hazard index, *Kp* dermal permeability coefficient (Wei et al. 2015)

**Table 5:** ILCR of cPAHs in the surface water samples from Buffalo River Estuary by dermal contact

PAHs	Kp (cm/h)	CSF	Children	Adults
Benzo(a)anthracene	8.10 × 10^−1^	0.73	2.83 × 10^−5^	4.79 × 10^−5^
Chrysene	8.10 × 10^−1^	0.073	6.94 × 10^−6^	1.18 × 10^−5^
Benzo(b)fluoranthene	1.20	0.73	1.39 × 10^−4^	2.36 × 10^−4^
Benzo(k)fluoranthene	–	0.73	–	–
Benzo(a)pyrene	1.20	7.3	9.87 × 10^−4^	1.67 × 10^−3^
Dibenzo(a,h)anthracene	2.70	7.3	2.35 × 10^−4^	3.98 × 10^−4^
Indeno(1,2,3-cd)pyrene	1.90	0.73	5.72 × 10^−4^	9.7 × 10^−4^
			1.97 × 10^−3^	3.34 × 10^−3^

*PAHs* polycyclic aromatic hydrocarbons, *CSF* cancer slope factor, *cPAHs* carcinogenic PAHs, *Kp* dermal permeability coefficient (Wang et al. 2009; CCME 2010; IARC 2010; Kumar et al. 2015; Wei et al. 2015; Benson et al. 2017; Błaszczyk et al. 2017)

It therefore could be inferred that Buffalo River Estuary water is unfit for swimming, because there is a chance that 1 in every 508 adults and 299 children who swim in the water are exposed to the risk of having cancer, respectively. This was in agreement with the Situation Assessment Report of the estuary earlier released (EOHCES 2016). It is worth mentioning that chances of adults’ exposure to the carcinogenic risk is higher than for children (Karyab et al. 2016). Hence, necessary caution should be taken by everyone using the water and regulatory bodies should as well ensure that the pollution of the aquatic environment is controlled to ensure the safety of human and aquatic lives.

## Conclusions

Although the total concentrations of PAHs found in the water and sediment of Buffalo River Estuary were above the regulatory limits, they were within the range of values reported from many other regions of the world. The two most polluted locations in the aquatic environment were E1 and E3. The levels of the pollutants were higher in summer than autumn and were predominantly from pyrolytic sources, especially from discharge of effluent from automobile and petrochemical industries, leachate from nearby landfill site, West Bank Hood Point domestic and industrial wastewater channels, vehicle emissions on the highways, harbour activities, as well as drains from urbanized subcatchments in the East London City Centre. Noncarcinogenic risk seems to be very unlikely in the water column, but the estimated carcinogenic risk by skin absorption was high. Similarly, TEQ and MEQ for benzo(a)pyrene and dibenzo(a,h)anthracene in the sediment were high, suggesting a possibility of carcinogenic and mutagenic hazards after frequent exposure. Adults seems to be more prone to higher level of risk than children. Therefore, necessary measures should be taken to address the level of contamination by the immediate users of the aquatic resources. Moreover, proper monitoring is needed in the area to safeguard human and aquatic lives in the area.
